# 

**Published:** 1897-05

**Authors:** 


					﻿Vol. XVII.
MAY, 1897.
NO. 5.
JBHE
HOMEOPATHIC PHYSICIAN,
A Monthly Journal of Homoeopathic Materia Medica
and Clinical Medicine.
“ Re is a freeman whom truth makes free, and all are slaves beside."
"Seek the Truth: come whence it may, cost what it will."
EDITED AND PUBLISHED BY
WALTER M. JAMES, M. D.
PHILADELPHIA :
Ko. 1231 LOCUST STREET.
Zj o n- u o n :
ALFRED HEATH & CO., Sole Agents ]for<| Great iBritain,
114 Ebury Street, Eaton 8quare.
Yearly Subscription, $2.50, invariably in advance. Single numbers, 25 etc.
Entered at the Philadelphia Post-Office ai Second-Class Mail Matter.
				

